# Directional Freezing Fabrication of RSF/PVA/MXene Aerogels: Layered Structure and High-Efficiency Electromagnetic Interference Shielding

**DOI:** 10.3390/ma19163549

**Published:** 2026-08-21

**Authors:** Peiyi Gao, Yanxiang Wang, Yingfan Li, Bohan Ding, Jinghe Guo, Yanru Yuan, Ziyi Xu, Dong Zhang, Lingyu Li, Xueqi Wang, Can Zhang, Chao Teng

**Affiliations:** 1 Carbon Fiber Engineering Research Center, School of Materials Science and Engineering, Shandong University, Jinan 250061, China; 2 College of Materials Science and Engineering, Qingdao University of Science and Technology, Qingdao 266042, China

**Keywords:** silk fibroin, MXene, aerogel, electromagnetic interference shielding, directional freeze-drying

## Abstract

The rapid development of wireless communication technologies and electronic devices has intensified electromagnetic interference (EMI) pollution, creating an urgent demand for lightweight and efficient shielding materials. In this work, RSF/PVA/MXene composite aerogels with ordered lamellar structures were fabricated by directional freeze-drying. Directional freeze-drying generated an ordered lamellar architecture, while RSF and PVA facilitated the homogeneous distribution of MXene nanosheets within the aerogel framework. Among the investigated samples, RPM-60 exhibited the maximum shielding effectiveness at 12.4 GHz, corresponding to a shielding efficiency of 99.45%, demonstrating the favorable MXene content for efficient EMI shielding at this frequency. The enhanced shielding performance was mainly attributed to the ordered lamellar structure, which promoted multiple internal reflections, together with conductive loss and interfacial/dipole polarization induced by MXene. These results demonstrate the potential of RSF/PVA/MXene composite aerogels as lightweight bio-based EMI shielding materials.

## 1. Introduction

With the rapid development of wireless communication technologies and the widespread use of electronic devices, electromagnetic waves have been extensively applied in communications, healthcare, aerospace, and defense. However, the increasing density and operating frequency of electronic devices have also caused serious electromagnetic interference (EMI) and electromagnetic pollution [1,2,3]. EMI can disturb the normal operation of precision electronic equipment, reduce signal transmission quality, and pose potential risks to human health and information security [4]. Conventional metal-based shielding materials exhibit excellent electrical conductivity and shielding effectiveness, but their high density, susceptibility to corrosion, poor flexibility, and processing difficulties limit their application in lightweight and flexible electronic devices [5,6]. In recent years, polymer-based conductive composites have attracted increasing attention because of their low density and easy processability [7]. Nevertheless, most conventional polymer matrices are derived from petroleum resources and suffer from poor biodegradability and environmental burdens. In addition, high filler content often leads to filler aggregation, which deteriorates the structural stability and EMI shielding effectiveness of the composites [8]. Therefore, developing lightweight, sustainable, and high-performance biomass-based EMI shielding materials is of great significance [9]. Among various electromagnetic frequency ranges, the X-band (8.2–12.4 GHz) is a key frequency band for research in the field of electromagnetic shielding materials. Since this band overlaps with the operating frequencies of numerous wireless communication devices and electronic components, testing electromagnetic shielding performance in this band provides a critical reference for practical applications of electromagnetic protection.

MXenes, as a class of emerging two-dimensional transition metal carbides, nitrides, and carbonitrides, have attracted considerable attention in EMI shielding since their first report in 2011 [10]. Owing to their metallic conductivity, unique two-dimensional layered structure, and abundant surface terminal groups, MXene nanosheets can construct conductive networks in composite systems and enhance conductive loss. Meanwhile, surface functional groups such as -O, -OH, and -F can contribute to interfacial polarization loss, thereby promoting electromagnetic wave attenuation [11,12,13]. For example, Li et al. constructed a highly ordered layered structure by ethanol-induced dispersion and orientation of MXene in aqueous epoxy resin, achieving excellent EMI shielding performance [14]. However, MXene nanosheets possess high surface energy and tend to aggregate or restack in polymer matrices, which reduces the effective utilization of conductive networks. Moreover, freestanding MXene films generally suffer from poor structural stability, limiting their practical application in lightweight lamellar shielding materials [15,16]. Therefore, selecting an appropriate polymer matrix to improve MXene dispersion, enhance interfacial interactions, and construct stable lamellar architectures is essential for developing high-performance MXene-based EMI shielding materials. An ideal polymer matrix for MXene-based EMI shielding composites should possess several characteristics, including strong interfacial interactions with MXene nanosheets, effective suppression of MXene aggregation, good processability for constructing stable architectures, and environmental sustainability.

Regenerated silk fibroin (RSF), obtained from natural silk after degumming, dissolution, and regeneration, is a renewable and biodegradable natural polymer with good biocompatibility [17,18]. The Silk-II crystalline structure of silk fibroin can provide favorable mechanical strength and structural stability [17]. More importantly, RSF contains abundant polar groups, including amino, hydroxyl, and carboxyl groups, which can interact with the surface terminal groups of MXene through hydrogen bonding [19]. These interactions are beneficial for suppressing MXene aggregation and enhancing the interfacial bonding between MXene nanosheets and the polymer matrix. Poly(vinyl alcohol) (PVA) was therefore introduced as a complementary polymer matrix component due to its water solubility, excellent film-forming ability, and abundant hydroxyl groups. The hydroxyl groups of PVA can form extensive hydrogen-bonding interactions with both RSF chains and MXene nanosheets, acting as a molecular bridge to enhance interfacial compatibility, inhibit MXene restacking, and stabilize the lamellar framework during directional freeze-drying. Therefore, the synergistic combination of RSF and PVA provides an effective polymer matrix for constructing lightweight and stable MXene-based aerogels.

Based on these considerations, RSF/PVA/MXene nanocomposite aerogels with long-range ordered lamellar structures were fabricated through a directional freeze-drying strategy. Unlike conventional RSF/MXene or PVA/MXene systems that mainly focus on improving electrical conductivity through MXene incorporation, this work emphasizes the construction of an ordered lamellar architecture to regulate electromagnetic wave propagation. The RSF/PVA matrix serves as the structural framework, while Ti_3_C_2_T_x_ MXene provides electrical conductivity and EMI shielding functionality. The effect of Ti_3_C_2_T_x_ MXene content (20, 40, 60, and 80 wt%) on the structure and EMI shielding performance of the composite aerogels was systematically investigated. The functional performance was evaluated based on EMI shielding effectiveness (SE_T_), shielding efficiency, and the contributions of absorption (A) and reflection (R) components. The ordered lamellar architecture generated during directional freezing is expected to enhance electromagnetic wave attenuation by extending the propagation pathway and increasing multiple internal reflections within the aerogel framework. Compared with our previous work on Janus RSF/PVA/MXene aerogels [20], which mainly focused on asymmetric structural design for reducing electromagnetic reflection, the present study constructs a uniform ordered lamellar architecture through directional freezing to regulate electromagnetic wave propagation and promote internal electromagnetic dissipation. This work aims to develop lightweight bio-based aerogels with ordered lamellar structures for efficient EMI shielding applications and to reveal the relationship between lamellar architecture and EMI shielding performance.

## 2. Materials and Methods

### 2.1. Materials

The materials used in this study are listed as follows. Bombyx mori cocoons were purchased from Ankang, Shaanxi, China. Ti_3_AlC_2_ MAX phase powder (≥98.0%, 400 mesh) was supplied by Jilin 11 Technology Co., Ltd., Jilin, China. Concentrated hydrochloric acid (HCl, 37%), lithium fluoride (LiF, 99%), anhydrous sodium carbonate (Na_2_CO_3_, ≥99.9%), calcium chloride (CaCl_2_, 96%), ethanol (C_2_H_5_OH, 99.5%), poly(vinyl alcohol) (PVA, Mw = 13,000–23,000, degree of hydrolysis = 98%) were obtained from Shanghai Macklin Biochemistry Technology Co., Ltd., Shanghai, China. Dialysis bags (molecular weight cut-off (MWCO) 8000–14,000 Da) were purchased from Shanghai Yuanye Technology & Biology Co., Ltd., Shanghai, China. All chemicals were used as received without further purification.

### 2.2. Preparation of Ti_3_C_2_T_x_ MXene Nanosheets

Ti_3_C_2_T_x_ MXene nanosheets were fabricated by selectively etching the Al layers from Ti_3_AlC_2_ MAX phase powder. Briefly, LiF (3.2 g) was dissolved in HCl solution (40 mL, 9 M) with continuous magnetic stirring for 30 min. Then, MAX phase powder (3.0 g) was added in small portions to the mixed solution, and the reaction was maintained at 40 °C for 48 h. To remove residual HCl and LiF, the resulting solid was washed repeatedly with deionized water and centrifuged at 3500 rpm for 5 min after each wash. This process was repeated until the supernatant pH reached 6. The clay-like sediment was then redispersed in an appropriate amount of deionized water, transferred into a gas-washing bottle, and purged with nitrogen for 20 min to prevent oxidation. After nitrogen purging, the dispersion was sonicated for 20 min and centrifuged at 3500 rpm for 20 min. The supernatant was collected, and the same cycle of nitrogen purging, sonication, and centrifugation was repeated two or three more times with the sediment. This yielded an aqueous dispersion of Ti_3_C_2_T_x_ MXene nanosheets with a concentration of 3 mg/mL.

### 2.3. Preparation of Regenerated Silk Fibroin Freeze-Dried Sponge

Bombyx mori cocoons (5 g) were cut into small pieces and degummed in 0.5 wt% Na_2_CO_3_ solution at 100 °C for 30 min. The degumming process was repeated 2–3 times to remove sericin. The degummed silk fibers were thoroughly rinsed with deionized water and dried at 40 °C. A ternary solvent consisting of CaCl_2_, C_2_H_5_OH, and H_2_O with a molar ratio of 1:2:8 was prepared. The dried silk fibers were added to the ternary solvent and dissolved at 80 °C for 40 min under stirring. The obtained solution was transferred into dialysis bags and dialyzed against deionized water for 72 h to remove residual salts and small molecules. The deionized water was replaced at 2, 4, 8, 12, 24, 36, and 48 h to ensure thorough removal of Ca^2+^. The dialyzed solution was subsequently freeze-dried and stored for further use.

### 2.4. Preparation of RSF/PVA/MXene Nanocomposite Aerogels

The as-prepared RSF sponge was dissolved in 10 wt% PVA aqueous solution to obtain an RSF/PVA mixed solution with a mass ratio of 1:1. Subsequently, under continuous stirring, aqueous dispersions of Ti_3_C_2_T_x_ MXene with mass fractions of 20%, 40%, 60%, and 80% were added to the RSF/PVA solution, respectively, to obtain homogeneous RSF/PVA/MXene precursor mixtures with different MXene contents. Each mixture was then poured into a custom-made polytetrafluoroethylene mold (3 cm × 3 cm × 3 cm) equipped with a copper template at the bottom. Liquid nitrogen was then slowly injected around the mold (freezing rate of −10 °C/min) to completely freeze the solution, followed by 72 h of freeze-drying under vacuum conditions (−70 °C, <2 Pa). The obtained aerogels were denoted as RPM-x, where x represents the weight percentage of Ti_3_C_2_T_x_ MXene in the total solid components. The MXene content was calculated based on the total mass of RSF, PVA, and Ti_3_C_2_T_x_ MXene.

### 2.5. Characterization

The morphology and microstructure of the samples were observed using a Schottky field emission scanning electron microscope (SEM, JSM 7610F, JEOL, Akishima, Japan and G-500, Zeiss, Oberkochen, Germany) equipped with an energy-dispersive X-ray spectroscopy detector (EDS) at an accelerating voltage of 10 kV. The morphology and structure of the MXene were further investigated by transmission electron microscopy (TEM, JEM-2100, JEOL, Akishima, Japan). XRD patterns were collected on an X-ray diffractometer (Cu Kα radiation, λ = 0.15406 nm, SmartLab, Rigaku, Tokyo, Japan) to determine the crystal structure. The functional groups were determined using a Fourier infrared spectrometer (FT-IR, TensorII, Bruker, Ettlingen, Germany). The chemical composition of the aerogel and MXene was determined by X-ray photoelectron spectroscopy (XPS, AXIS Supra, Shimadzu, Kyoto, Japan and Nexsa, Thermo Fisher Scientific, Hillsboro, OR, USA) using an Al Kα anode. Electromagnetic parameters were measured over 8.2–12.4 GHz using a vector network analyzer (VNA, Agilent Technologies N5244A, Keysight Technologies, Santa Rosa, CA, USA). The specimen was clamped between the waveguide ports. The samples were cut into rectangular prism specimens with dimensions of 22.86 mm × 10.16 mm × 3 mm and placed between the waveguide flanges. The scattering parameters S_11_ (reflection) and S_21_ (transmission) were recorded to calculate the reflection (R), absorption (A), and transmission (T) coefficients. The total EMI SE (SE_T_), absorption SE (SE_A_), and reflection SE (SE_R_) were determined from the following equations [21]:

(1) $$R={\left|S_{11}\right|}^{2}={\left|S_{22}\right|}^{2}$$

(2) $$T={\left|S_{12}\right|}^{2}={\left|S_{21}\right|}^{2}$$

(3) A=1−R−T

(4) $${SE}_{R}=-10\log\left(1-R\right)=-10log(1-{\left|S_{11}\right|}^{2})$$

(5) $${SE}_{A}=-10\log\left(\frac{T}{1-R}\right)=-10log(\frac{{\left|S_{21}\right|}^{2}}{1-{\left|S_{11}\right|}^{2}})$$

(6) $${SE}_{T}={SE}_{R}+{SE}_{A}$$

## 3. Results and Discussion

**Figure 1:**
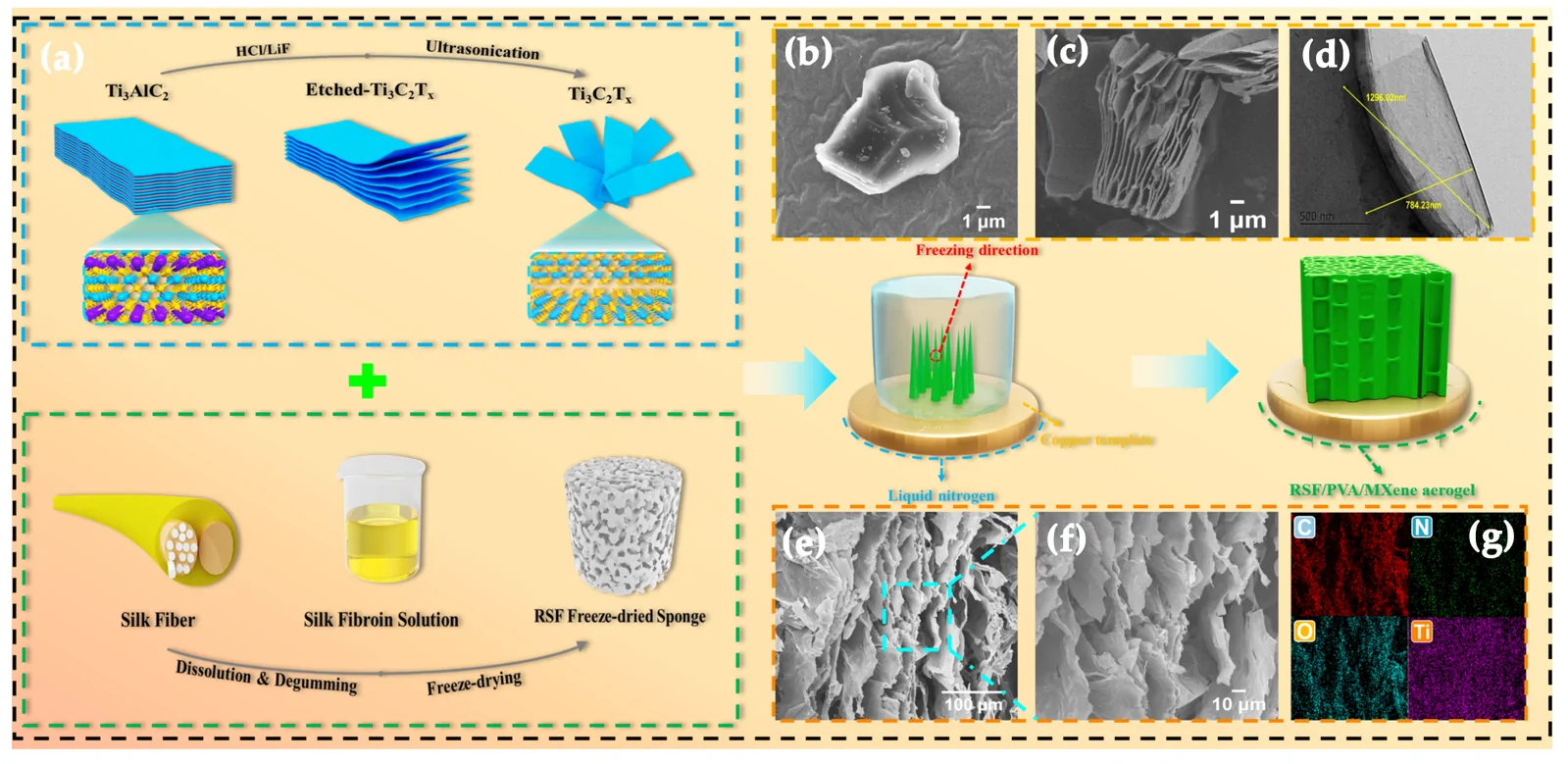
(**a**) Schematic illustration of the fabrication of RSF/PVA/MXene composite aerogels; (**b**) SEM image of Ti_3_AlC_2_ MAX phase; (**c**) SEM image of multilayer Ti_3_C_2_T_x_ MXene; (**d**) TEM image of delaminated Ti_3_C_2_T_x_ MXene nanosheets; (**e**,**f**) cross-sectional SEM images of the RSF/PVA/MXene composite aerogel; (**g**) EDS elemental mapping images of the RSF/PVA/MXene composite aerogel.

Figure 1a illustrates the fabrication process of the RSF/PVA/MXene composite aerogels. First, Ti_3_C_2_T_x_ MXene nanosheets were prepared via an HCl/LiF etching method [22]. Subsequently, RSF, PVA, and MXene at different concentrations were uniformly mixed, and a composite aerogel with a layered structure was fabricated through directional freeze-drying [23,24].

Silkworm cocoons (Figure S1a) consist of several bundles of natural silk fibers wound together in a random manner. After degumming, the silk fibers (Figure S1b) exhibit a smooth and fine surface, with the fibers intertwined and distributed randomly. The freeze-dried RSF exhibits interconnected fibrous and lamellar structures, indicating that a continuous three-dimensional network structure was formed during the freeze-drying process (Figure S1c,d). The precursor MAX phase (Ti_3_AlC_2_) (Figure 1b) has an overall block-like structure, with a dense layered structure observable internally. The surface exhibits minor wrinkles, but no obvious defects, and it possesses clear and distinct edges. The multilayer Ti_3_C_2_T_x_ MXene (Figure 1c) exhibits a distinct “accordion-like” structure, which results from the selective etching of Al layers, leading to an increased interlayer spacing. Figure 1d shows a TEM image of Ti_3_C_2_T_x_ MXene, revealing that the single-layer Ti_3_C_2_T_x_ nanosheets are essentially transparent and free of obvious defects, indicating that the MXene nanosheets were successfully exfoliated.

Figure 1e,f show the layered structure within the RSF/PVA/MXene composite aerogel. We attribute this layered structure to the oriented growth and templating effect of ice crystals during the direct freezing process, while RSF and PVA serve as a skeletal support. During the freeze-drying of the RSF/PVA/MXene mixed solution, PVA acts as a “bridge,” and its hydrogen bonding interactions with both RSF and Ti_3_C_2_T_x_ MXene help stabilize the lamellar structure and promote uniform MXene dispersion. Different from the previously reported Janus RSF/PVA/MXene aerogels, the present material exhibits a uniform lamellar architecture throughout the aerogel framework. Such continuous lamellar channels provide extended pathways for electromagnetic wave propagation and promote multiple internal reflections, contributing to enhanced electromagnetic attenuation. Meanwhile, Figure 1g shows the elemental distribution of the RSF/PVA/MXene composite aerogel, in which C, N, O, and Ti are uniformly distributed, indicating that Ti_3_C_2_T_x_ MXene is well dispersed within the system.

**Figure 2:**
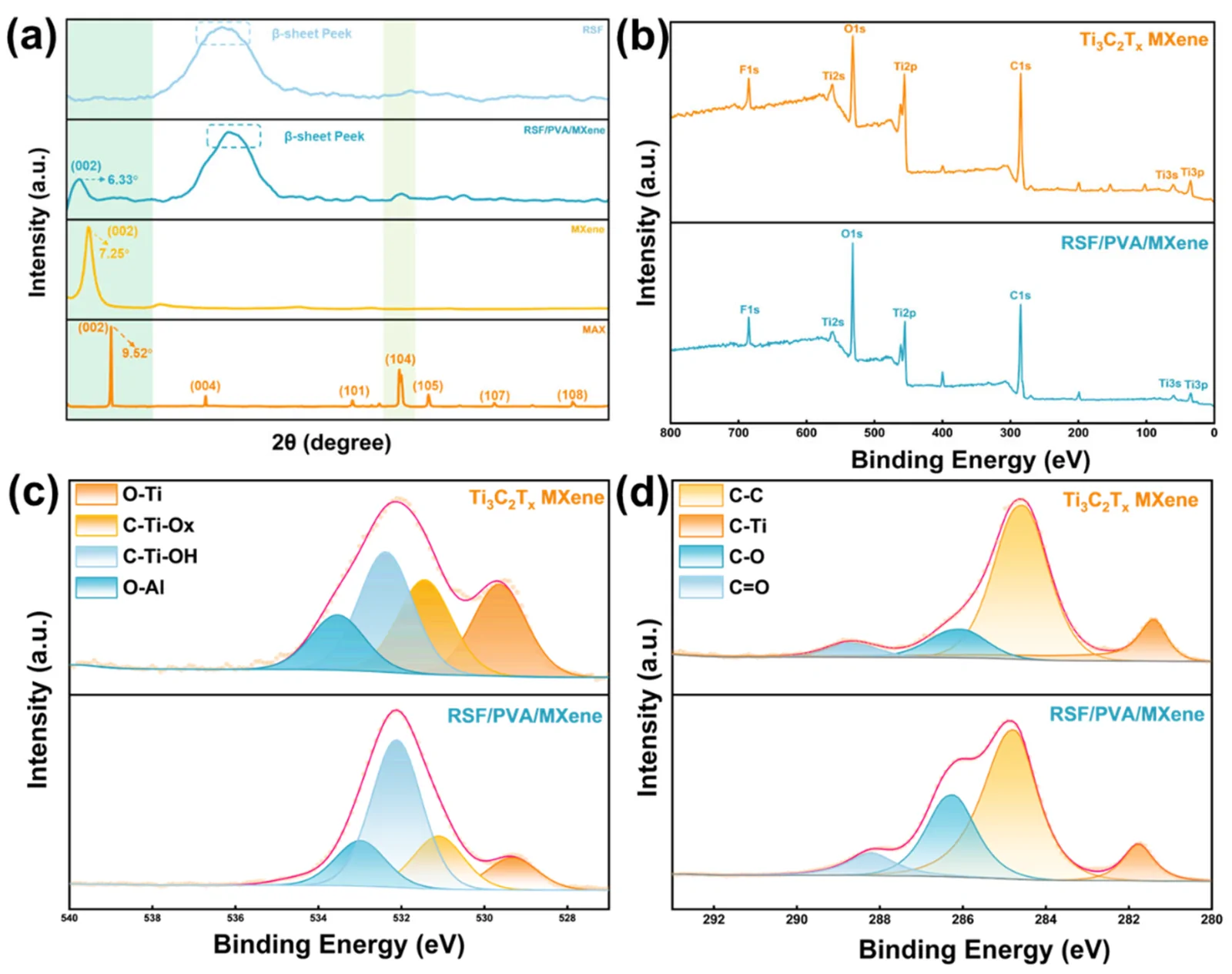
(**a**) XRD patterns of RSF, RSF/PVA/MXene composite aerogel, MXene, and MAX phase. XPS spectra of MXene and the RSF/PVA/MXene composite aerogel: (**b**) survey spectrum, (**c**) O 1 s, (**d**) C 1 s.

Figure S1f compares the XRD patterns of Ti_3_AlC_2_ and Ti_3_C_2_T_x_ MXene. Compared with the Ti_3_AlC_2_ MAX phase, the (002) diffraction peak of Ti_3_C_2_T_x_ MXene shifted from 9.52° to 7.25°, indicating an enlarged interlayer spacing after etching. Meanwhile, the characteristic peaks of Ti_3_AlC_2_, such as (004), (101), and (104), disappeared, suggesting the effective removal of Al layers and the successful preparation of Ti_3_C_2_T_x_ MXene [25,26]. Figure 2a shows, from top to bottom, the XRD patterns of RSF, RSF/PVA/MXene aerogel, Ti_3_C_2_T_x_ MXene, and Ti_3_AlC_2_. The broad peak near 20° is mainly attributed to the β-sheet crystalline structure of RSF, although the crystalline diffraction of PVA may also contribute to this region. As shown in the figure, the XRD pattern of RSF/PVA/MXene retains the (002) characteristic peak, which has shifted from 7.25° to 6.33°. According to Bragg’s law, the shift of the (002) peak toward lower angles indicates a further increase in the interlayer spacing of MXene. This is due to the insertion of RSF and PVA between MXene layers, while hydrogen bonding inhibits the restacking of MXene. Furthermore, the characteristic peaks corresponding to the β-sheet-related diffraction feature of RSF around 20° were retained, indicating the successful preparation of the RSF/PVA/MXene composite aerogel [20].

Figure 2b shows the full spectra of Ti_3_C_2_T_x_ MXene and the RSF/PVA/MXene composite aerogel. The full spectrum shows that peaks from elements such as C, Ti, O, and F can be observed in the Ti_3_C_2_T_x_ MXene, while peaks from elements such as C, N, O, and Ti appear simultaneously in the RSF/PVA/MXene composite aerogel, indicating that MXene has been successfully incorporated into the RSF/PVA matrix. Figure 2c shows the O 1s spectrum, in which peaks associated with O-Ti, C–Ti-O_x_, and C-Ti-OH bonds are observed, indicating the presence of oxygen-containing functional groups on the MXene surface [27,28]. Combined with characteristic peaks such as C–Ti in the C 1s spectrum (Figure 2d), this further confirms the presence of Ti_3_C_2_T_x_ MXene. No obvious new covalent-bond-related peaks were observed in the RSF/PVA/MXene composite aerogel, indicating that RSF/PVA and MXene are primarily bound through noncovalent interactions such as hydrogen bonding [29]. To further investigate the intermolecular interactions within the composite aerogel, FTIR spectra were analyzed (Figure S3). As shown in Figure S3, the RSF/PVA/MXene composite aerogel exhibits the characteristic absorption bands of RSF, including the amide I, amide II, and amide III bands located at approximately 1631, 1512, and 1396 cm^−1^, respectively. Meanwhile, the peak at approximately 557 cm^−1^ can be assigned to the Ti–C vibration of Ti_3_C_2_T_x_ MXene, confirming the successful incorporation of MXene into the composite aerogel. Compared with pristine RSF, the variations in the amide I and amide II bands after introducing PVA and MXene indicate changes in the hydrogen-bonding environment and intermolecular interactions within the composite system. These results suggest that noncovalent interactions, including hydrogen bonding, contribute to the improved interfacial compatibility between RSF/PVA and MXene.

**Figure 3:**
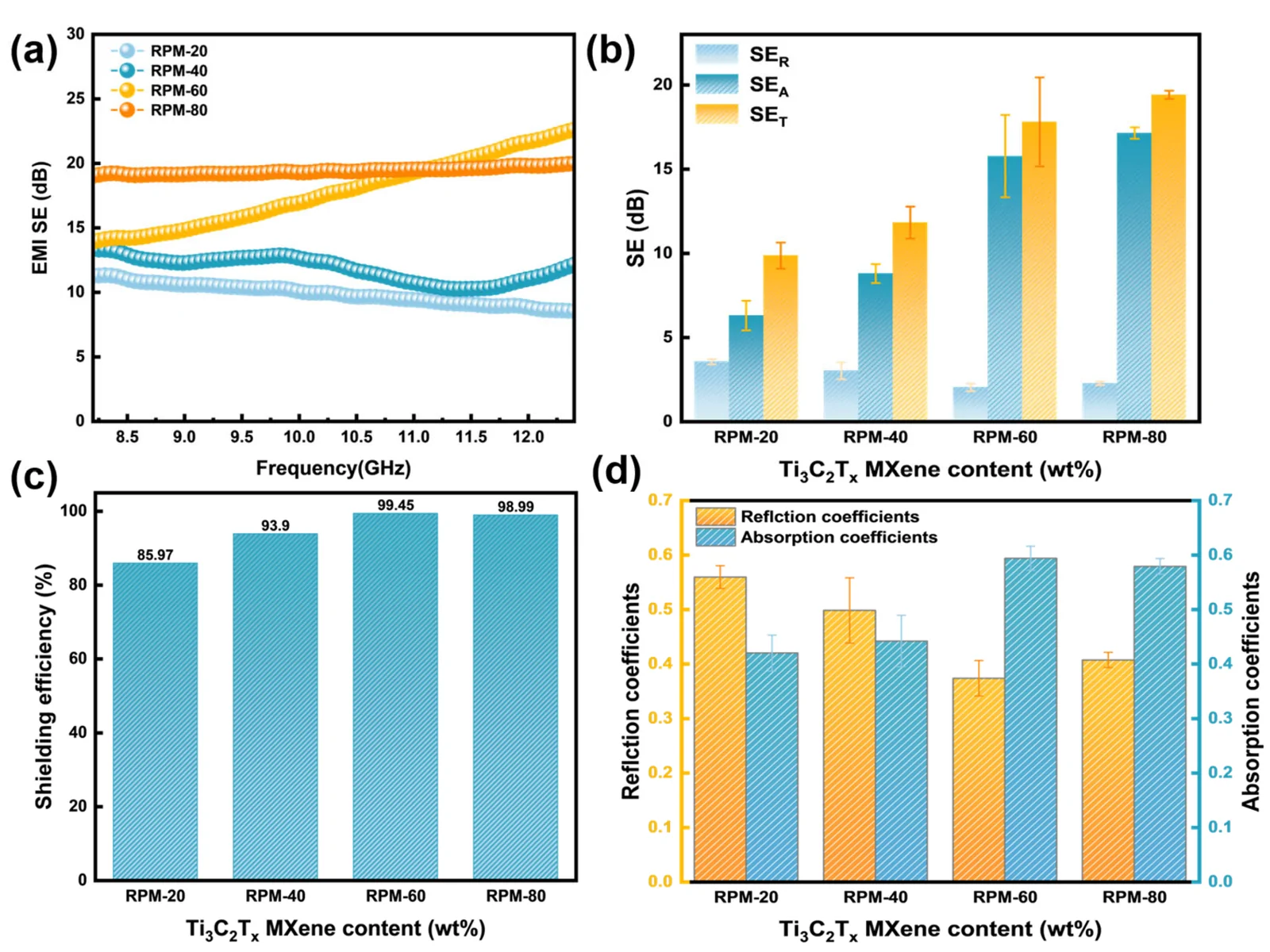
Electromagnetic interference shielding performance of RSF/PVA/MXene composite aerogels. (**a**) EMI SE of the RSF/PVA/MXene composite aerogels with different MXene contents in the X-band. (**b**) Average SE_T_, SE_A_, and SE_R_ values in the X-band of the RSF/PVA/MXene composite aerogels. Error bars represent the standard deviations calculated from the corresponding frequency-dependent data points. (**c**) Shielding efficiency of the RSF/PVA/MXene composite aerogels with different MXene contents at 12.4 GHz. (**d**) R and A coefficients in the X-band. Error bars represent the standard deviations calculated from the corresponding frequency-dependent data points [30,31].

The EMI SE of RSF/PVA/MXene composite aerogels with different MXene contents was investigated using a vector network analyzer, as shown in Figure 3. Calculations show that the maximum electromagnetic shielding values of the RSF/PVA/MXene composite aerogels at Ti_3_C_2_T_x_ MXene contents of 20%, 40%, 60%, and 80% are 8.53 dB, 12.16 dB, 22.62 dB, and 19.98 dB, respectively. The EMI shielding effectiveness was enhanced with increasing Ti_3_C_2_T_x_ MXene content, although the influence of MXene content was frequency-dependent. RPM-60 achieved the highest SE value of 22.62 dB at 12.4 GHz among all samples, and the shielding efficiency reached 99.45% (Figure 3c), meeting the basic requirement for common commercial EMI shielding applications. The improvement from RPM-20 to RPM-60 is mainly attributed to the enhanced conductive pathways provided by Ti_3_C_2_T_x_ MXene nanosheets. However, when the MXene content was further increased to 80 wt%, excessive MXene nanosheets may undergo partial stacking, which could reduce their effective dispersion within the RSF/PVA matrix and weaken the synergistic contribution of the MXene network and ordered lamellar architecture, leading to a decrease in EMI SE. Furthermore, to better understand the EMI shielding mechanism, by analyzing the measured total electromagnetic shielding (SE_T_ = SE_A_ + SE_R_), absorption (SE_A_), and reflection (SE_R_) components (Figure 3b), we can observe that the SE_T_ of the RSF/PVA/MXene composite aerogel with a Ti_3_C_2_T_x_ MXene content of 20 wt% is relatively low. At the same time, SE_A_ contributes more to EMI SE than SE_R_, and the higher Ti_3_C_2_T_x_ MXene content indicates that the absorption mechanism plays a major role; however, relying solely on SE_A_ and SE_R_ to explain the material’s absorption or reflection mechanisms is not sufficiently convincing. Based on this, we further evaluated the interaction between electromagnetic waves and the aerogel during transmission by analyzing the reflection (R), absorption (A), and transmission (T) coefficients of the RSF/PVA/MXene composite aerogel using the obtained S-parameters. As shown in Figure 3d, as the MXene content increases, the A coefficient rises significantly, while the R coefficient decreases overall, indicating that the mode of electromagnetic wave attenuation gradually shifts from surface reflection to internal absorption. Among them, the RPM-60 sample exhibits the highest A value and the lowest R value, indicating that it possesses more pronounced absorption-dominated shielding characteristics. These results indicate that the EMI shielding mechanism gradually shifts from surface reflection to internal absorption with increasing MXene content, and the optimized RPM-60 sample shows absorption-dominated shielding characteristics. This absorption-dominated shielding behavior differs from the low-reflectivity strategy achieved by the Janus architecture in our previous study, indicating that the present ordered lamellar structure provides an alternative pathway for efficient EMI attenuation. As shown in Figure S2, the optimized RSF/PVA/MXene aerogel exhibits an EMI SE of 22.62 dB, which is comparable to or higher than those of several reported MXene-based or PVA-based composites, further demonstrating its promising EMI shielding performance. To further understand the relationship between structural characteristics and EMI shielding performance, the apparent densities of the prepared aerogels were calculated and summarized in Table S1. The densities of RPM-20, RPM-40, RPM-60, and RPM-80 were 0.0107, 0.0098, 0.0079, and 0.0076 g cm^−3^, respectively, demonstrating the ultralight characteristics of the prepared aerogels. Based on the measured density values, the specific shielding effectiveness (SSE) was further calculated to evaluate the lightweight shielding capability of the aerogels. As shown in Figure S4, all samples exhibited high SSE values throughout the X-band frequency range. Among them, RPM-60 showed the highest SSE value, reaching approximately 2863 dB cm^3^ g^−1^ at 12.4 GHz, indicating its excellent lightweight EMI shielding capability. However, the enhanced EMI shielding performance cannot be solely explained by density variation but rather originates from the synergistic contribution of conductive Ti_3_C_2_T_x_ MXene nanosheets and the ordered lamellar architecture. Considering that thickness is an important factor affecting EMI shielding performance, the thickness-normalized SSE values (SSE/t) were further calculated and are presented in Figure S5. Since all samples possessed an identical thickness of approximately 3 mm, the variation trend of SSE/t remained consistent with that of SSE, further demonstrating that the shielding performance comparison was not affected by thickness variation.

**Figure 4:**
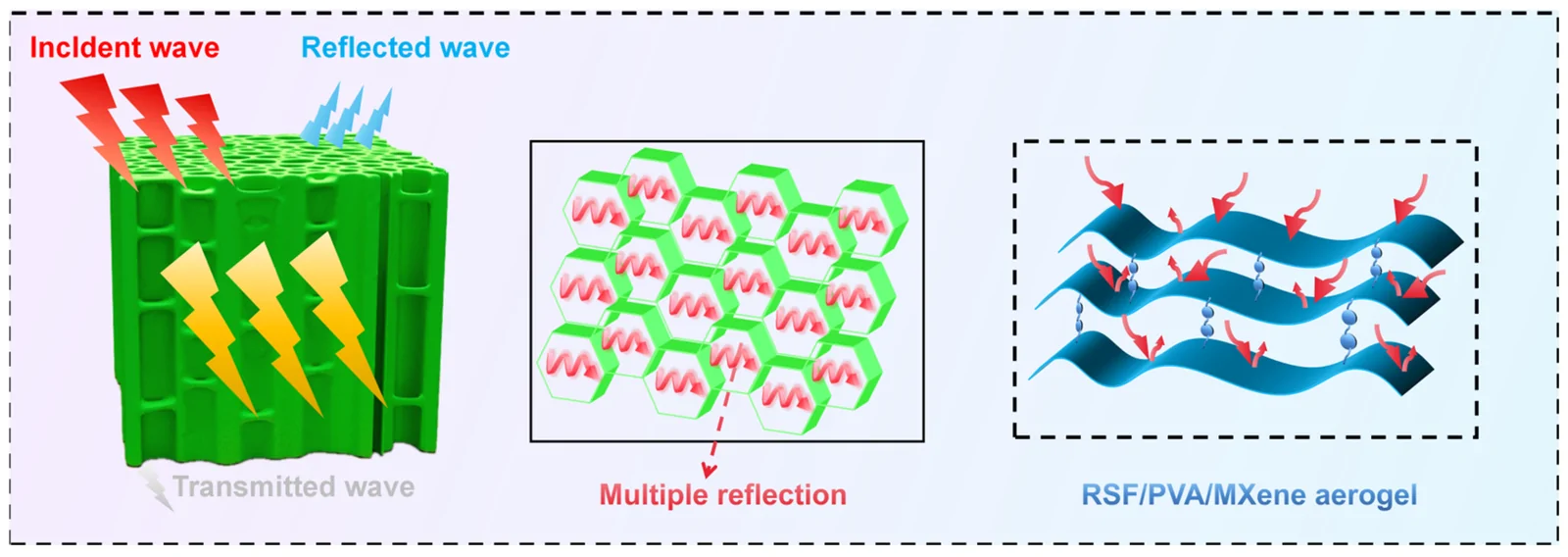
Schematic diagram of the electromagnetic shielding mechanism of the RSF/PVA/MXene composite aerogel.

Based on the above results, the EMI shielding mechanism of the RSF/PVA/MXene composite aerogel is proposed in Figure 4. During directional freezing, ice crystals grow along the temperature gradient and act as templates to induce the formation of aligned lamellar channels. The ordered lamellar channels generated by ice templating provide repeated interfaces for electromagnetic wave scattering and reflection, thereby increasing the propagation path of electromagnetic waves within the aerogel. Ti_3_C_2_T_x_ MXene nanosheets contribute to electromagnetic attenuation through conductive loss and polarization loss. Meanwhile, PVA serves as a hydrogen-bonding bridge by interacting with both RSF molecular chains and MXene surface terminal groups, which improves interfacial compatibility and promotes uniform MXene dispersion.

## 4. Conclusions

In this work, RSF/PVA/MXene composite aerogels with ordered lamellar structures were successfully fabricated through directional freeze-drying using Bombyx mori cocoons as the biomass source. PVA acted as a hydrogen-bonding bridge among RSF, PVA, and MXene surface terminations, which improved interfacial compatibility, suppressed MXene agglomeration, and stabilized the lamellar channels. The EMI shielding performance was strongly dependent on MXene loading and measurement frequency. The optimized RPM-60 aerogel achieved a maximum SE_T_ of 22.62 dB at 12.4 GHz, corresponding to a shielding efficiency of 99.45%, meeting the basic requirement for common commercial EMI shielding applications. Power coefficient analysis indicated that the EMI shielding behavior was mainly dominated by absorption. The enhanced shielding performance originated from the synergistic effects of multiple internal reflections within the ordered lamellar channels, ohmic loss induced by the conductive MXene nanosheets, and polarization loss arising from MXene surface terminations and heterogeneous interfaces. This work provides new insights into the role of ordered lamellar architectures in regulating electromagnetic wave propagation and attenuation in biomass-based MXene aerogels. Although the present work demonstrates excellent EMI shielding performance, the mechanical properties and long-term structural stability of the aerogels require further systematic investigation for practical applications.

## Data Availability

The original contributions presented in this study are included in the article/Supplementary Materials. Further inquiries can be directed to the corresponding authors.

## Supplementary Materials

The following supporting information can be downloaded at: https://www.mdpi.com/article/10.3390/ma19163549/s1. Figure S1: (a) SEM image of the Bombyx mori cocoon surface. (b) SEM image of degummed silk fibers. (c) SEM image of RSF. (d) High-resolution SEM image of RSF. (e) Schematic diagram of the silk fibroin fiber structure. (f) XRD patterns of MXene and MAX phase. Figure S2: Comparison of the EMI shielding effectiveness of the RSF/PVA/MXene aerogel with representative MXene-based and PVA-based composite materials reported in the literature [15,32,33,34,35]. Figure S3: FT-IR spectroscopy of RSF and RSF/PVA/MXene composite aerogels. Figure S4: SSE of RSF/PVA/MXene composite aerogels with different Ti_3_C_2_T_x_ MXene contents in X-band. Figure S5: SSE/t of RSF/PVA/MXene composite aerogels with different Ti_3_C_2_T_x_ MXene contents in X-band. Table S1: Density of RSF/PVA/MXene composite aerogels with different Ti_3_C_2_T_x_ MXene contents.
