# Supplementary material for: Directional Freezing Fabrication of RSF/PVA/MXene Aerogels: Layered Structure and High-Efficiency Electromagnetic Interference Shielding
Source: Materials (Basel). 2026 Aug 21;19(16):3549. doi: 10.3390/ma19163549 (PMC13514975; doi:10.3390/ma19163549)
Supplement: Supplementary file 1 [file materials-19-03549-s001.zip › materials-4350554-supplementary.pdf]

**SUPPLEMENTARY MATERIALS**

# Directional Freezing Fabrication of RSF/PVA/MXene Aerogels: Layered Structure and High-Efficiency Electromagnetic Interference Shielding


**Peiyi Gao [1], Yanxiang Wang [1,*], Yingfan Li [1], Bohan Ding [1], Jinghe Guo [1], Yanru Yuan [1], Ziyi Xu [1], Dong Zhang [1], Lingyu Li [1], Xueqi Wang [1], Can Zhang [1] and Chao Teng [2,*]**

[1] Carbon Fiber Engineering Research Center, School of Materials Science and Engineering, Shandong University, Jinan 250061, China

[2] College of Materials Science and Engineering, Qingdao University of Science and Technology, Qingdao 266042, China

* Correspondence: wyx079@sdu.edu.cn (Y.W.); tengchao2011@126.com (C.T.)


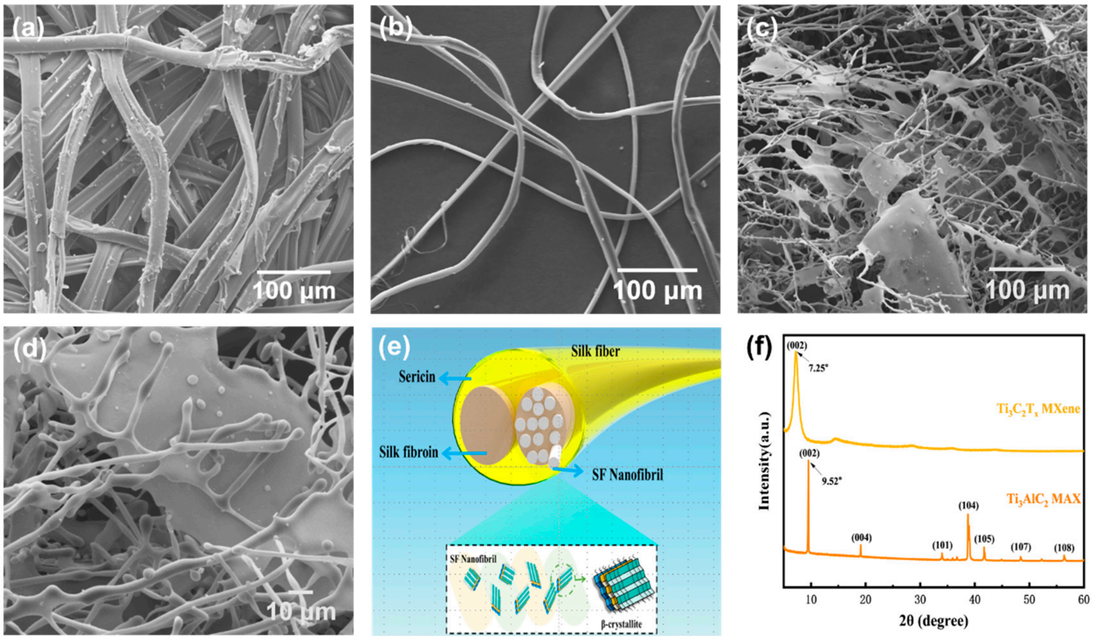


**Figure S1.** (a) SEM image of the Bombyx mori cocoon surface. (b) SEM image of degummed silk fibers. (c) SEM image of RSF. (d) High-resolution SEM image of RSF. (e) Schematic diagram of the silk fibroin fiber structure. (f) XRD patterns of MXene and MAX phase.

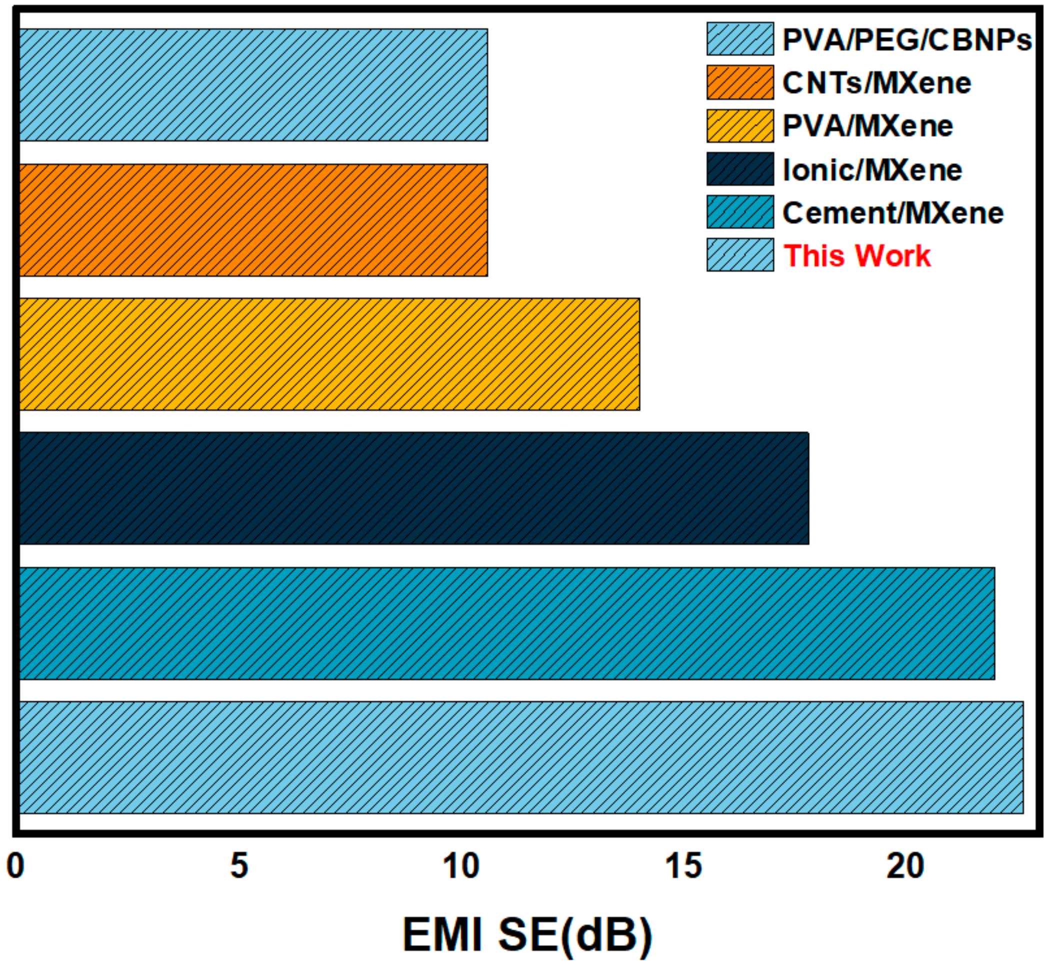


**Figure S2.** Comparison of the EMI shielding effectiveness of the RSF/PVA/MXene aerogel with representative MXene-based and PVA-based composite materials reported in the literature.[1–5]

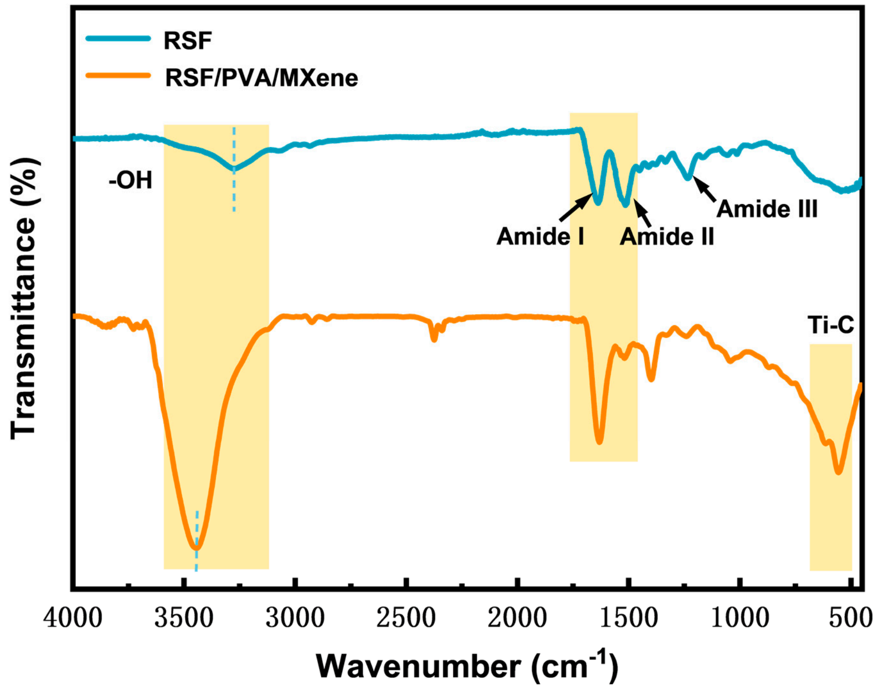


**Figure S3.** FT-IR spectroscopy of RSF and RSF/PVA/MXene composite aerogel s.

| Sample | $Ti_3C_2T_x$ MXene content (wt%) | Density (g $cm^{-3}$) |
|---|---|---|
| RPM-20 | 20 | 0.0107 |
| RPM-40 | 40 | 0.0098 |
| RPM-60 | 60 | 0.0079 |
| RPM-80 | 80 | 0.0076 |

**Table S1.** Density of RSF/PVA/MXene composite aerogels with different $Ti_3C_2T_x$ MXene contents.

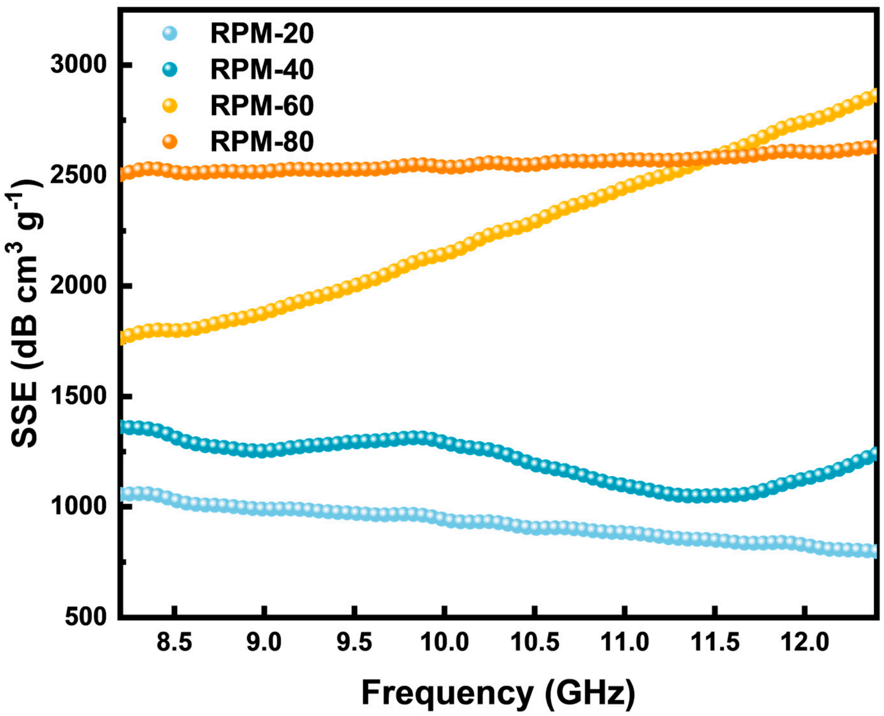


**Figure S4.** SSE of RSF/PVA/MXene composite aerogels with different $Ti_3C_2T_x$ MXene contents in X-band.

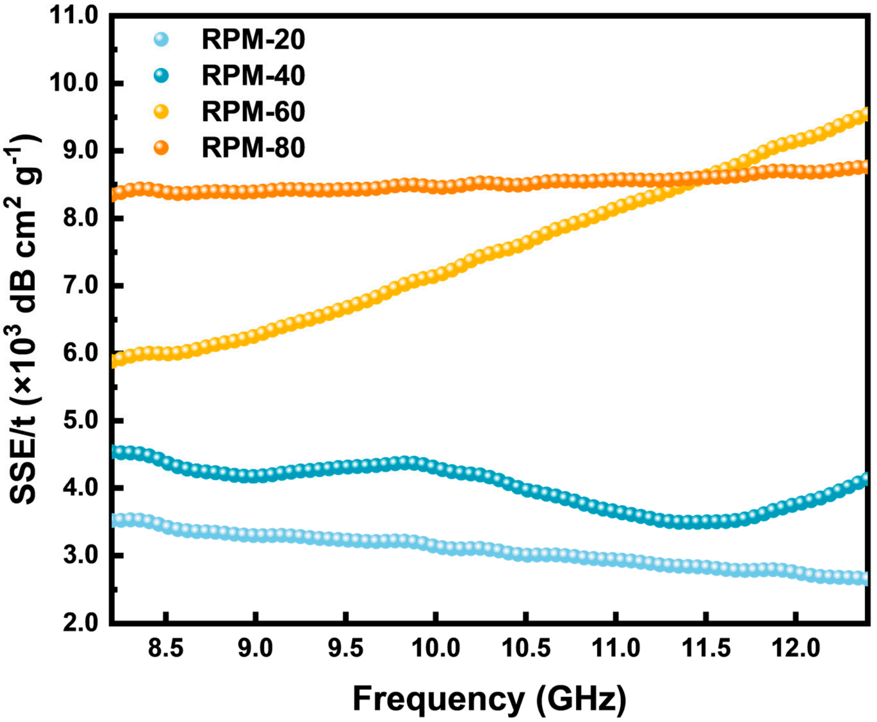


**Figure S5.** SSE/t of RSF/PVA/MXene composite aerogels with different $Ti_3C_2T_x$ MXene contents in X-band.

## Reference


1. Rani, P.; Ahamed, M.B.; Deshmukh, K. Dielectric and Electromagnetic Interference Shielding Properties of Carbon Black Nanoparticles Reinforced PVA/PEG Blend Nanocomposite Films. Mater. Res. Express 2020, 7, 064008, doi:10.1088/2053-1591/ab9853.
2. Chang, R.; Gao, H.; Hao, P.; Qu, H.; Xu, J.; Ma, J. Hierarchically Porous MXene/Carbon Nanotube/Epoxy Composites for Effective Electromagnetic Interference Shielding and Flame Retardancy. ACS Appl. Nano Mater. 2024, 7, 3168–3178, doi:10.1021/acsanm.3c05512.
3. Radhika, T.; Vasanthakumari, K.G.; Shajahan, M. PVA/MXene and PVA-Chitosan/MXene Flexible Films for EMI Shielding Applications. J. Mater. Sci.: Mater. Electron. 2025, 36, 2058, doi:10.1007/s10854-025-16146-3.
4. Chen, L.; Geng, C.; Yu, S.; Xu, H.; Bao, F.; Yuan, Z.; Zhang, X.; Wang, L. Dual-Conduction Network in Ionic/MXene Hydrogels for Advanced Sensing and Electromagnetic Shielding. J. Polym Sci. 2026, 64, 522–532, doi:10.1002/pol.20251014.
5. Wei, F.; Liu, Q.; Liu, H.; Wang, G.; Li, M.; Li, M.; Liu, X. Advanced $Ti_3C_2T_x$ MXene-Modified Cement Nanocomposites toward High Efficiency Electromagnetic Functional Materials for Green Buildings. Int. J. Appl. Ceram. Technol. 2023, 20, 251–260, doi:10.1111/ijac.14202.
